# *Epilepsia Open* – February 2024 Announcements

**DOI:** 10.1002/epi4.12911

**Published:** 2024-02-04

## 1 ILAE Congresses

17th Latin American Summer School on Epilepsy (LASSE XVII)

16–24 February 2024

São Paulo, Brazil

4th ILAE School on Neuropsychology in Epilepsy

3–8 March 2024

Lyon, France

6th ILAE School on EEG in the First Year of Life: From newborn to toddler

25–28 March 2024

Cambridge, UK & Online

ILAE School on Neuroimaging 2024 (AMIE & SuSIE 2024)

15–18 May 2024

Potsdam, Berlin & Online

XIII Congreso Latinoamericano de Epilepsia

15–18 June 2024

Santo Domingo, República Dominicana

6th ILAE School on Advanced EEG and Epilepsy in Dianalund

20–27 July 2024

Dianalund, Denmark

15th European Epilepsy Congress

7–11 September 2024

Rome, Italy

14th International Summer School for Neuropathology and Epilepsy Surgery

19–22 September 2024

Erlangen, Germany

15th Asian & Oceanian Epilepsy Congress

November 2024

New Delhi, India

36th International Epilepsy Congress

30 August–3 September 2025

Lisbon, Portugal

## 2 ILAE Webinars

ILAE‐Asia & Oceania YES Webinar

8 February 2024

Virtual Ketogenic Dietary Therapies Workshop for Africa

10 February 2024

Updates to mhGAP Guidelines

12 February 2024

La Journée internationale de l’épilepsie

13 February 2024

ILAE e‐Forum: Optimal models of care during transition from childhood to adulthood

16 February 2024, 13:30 ‐ 15:00 UTC

ILAE‐YES Eastern Mediterranean Journal Club

19 February 2024

Epilepsy Educational Webinar

23 February 2024

Climate Change Commission Webinar

24 March 2024

Living with Epilepsy: Conversation between a patient and a psychologist

25 April 2024

## 3 Other Congresses

Alpine EEG Course

25–28 February 2024

Oberstdorf, Germany

Kuopio Epilepsy Symposium

13–15 March 2024

Kuopio, Finland

Young Epilepsy Symposium Switzerland 2024

21 March 2024

Berne, Switzerland

9th London Innsbruck Colloquium on Status Epilepticus and Acute Seizures

8–10 April 2024

London, United Kingdom

Paros Epilepsy

22–26 April 2024

Paros, Greece

Seventeenth Eilat Conference on New Antiepileptic Drugs and Devices (EILAT XVII)

5–8 May 2024

Madrid, Spain

13th World Congress for NeuroRehabilitation

22–25 May 2024

Vancouver, Canada

Third International Workshop on High Frequency Oscillations in Epilepsy

23–25 May 2024

New York, NY, USA

SNS Annual Meeting 2024

6–7 June 2024

Basel, Switzerland

62. Jahrestagung der Deutschen Gesellschaft für Epileptologie

12–15 June 2024

Offenburg, Germany

10th Congress of the European Academy of Neurology

29 June–2 July 2024

Helsinki, Finland

20th Advanced San Servolo Epilepsy Course

21 July–1 August 2024

San Servolo (Venice), Italy

10th Eilat International Educational Course: Pharmacological Treatment of Epilepsy

15–20 September 2024

Jerusalem, Israel

Epilepsy Society of Australia Annual Scientific Meeting 2024

6–8 November 2024

Hobart, Tasmania, Australia

